# Descriptive review of internet-based cognitive behavior therapy on anxiety-related problems in children under the circumstances of COVID-19

**DOI:** 10.1186/s13030-021-00233-y

**Published:** 2022-01-10

**Authors:** Kentaro Shirotsuki, Nagisa Sugaya, Mutsuhiro Nakao

**Affiliations:** 1 Faculty of Human Sciences, Musashino University, 3-3-3, Ariake, Koto-ku, Tokyo, 135-8181 Japan; 2 Unit of Public Health and Preventive Medicine, School of Medicine, Yokohama City, Japan; 3 Department of Psychosomatic Medicine, School of Medicine, International University of Health and Welfare, Chiba, Japan

**Keywords:** Internet, Cognitive behavioral therapy, Anxiety, Child, COVID-19

## Abstract

**Background:**

COVID-19 continues to have a global impact and has yet to converge. Behavioral restrictions in daily life are widespread, forcing changes to the behavioral patterns of people. Significant changes have also occurred in children’s lives, raising concerns about mental health. The same is true for anxiety symptoms.

**Research:**

In this paper, we described the COVID-19 pandemic effects on mental health, summarized Internet-based cognitive behavioral therapy (ICBT) as an applicability of cognitive behavioral therapy (CBT) for COVID-19, and summarized ICBT’s current state as a response for anxiety. An overview of previous intervention studies, including randomized controlled trials (RCTs) on ICBT, showed that many studies were highly effective against anxiety symptoms. Furthermore, regarding the follow-up of ICBT’s intervention effect, long-term effect maintenance was also clarified. It was likewise pointed out that ICBT may be used in the future since it is beneficial for children’s anxiety symptoms in telemedicine.

**Conclusions:**

Based on these results from previous studies, we discuss ICBT’s applicability during the COVID-19 pandemic. Additionally, future measures and prospects for children’s mental health during the pandemic are discussed in this study.

## Background

The effects of the coronavirus disease 2019 (COVID-19) have been long-lasting. COVID-19’s chronic effects have brought about major changes in people’s social lives around the world. Ensuring social distancing and reducing interpersonal relationships are needed worldwide to combat the present COVID-19 pandemic. It has various effects such as increased stress, heightened anxiety, and worsening depression [1, 2]. Moreover, it has chronic effects on children’s social life and mental health.

According to UNESCO’s report, increased COVID-19 infection rates caused school closures around the world. Over 1.5 billion students across all education levels were affected by April 2020. Particularly, over 1.2 billion of them were in the Asia-Pacific region [3]. Moreover, the report suggested that 93% of the countries across the world and 90% of countries in the Asia-Pacific fully or partially closed their schools. For example, students in the Asia-Pacific region lost an average of 101 study days (0.6 years) due to school closures. Thus, their estimated secondary education completion rate is only 76%. Almost two-thirds of the 46 countries in the Asia-Pacific region developed online teaching and learning programs during the COVID-19 pandemic. Over 41% of the countries all over the world and 26% in the Asia-Pacific region used television and radio broadcasting, social media platforms such as Facebook and YouTube, and offline programs. Around 22% of the countries also used various storage and distribution means (i.e., e-books, DVDs) as a medium of instruction. It should be noted that most countries have deployed more than one platform or method to continue students’ learning activities [4]. Students can significantly reduce their learning losses if they can use appropriate and effective tools as solutions for this problem. These include online platforms for learning. Apart from the potential inequality caused by the digital divide and distance learning practices, school closures may also cause disruptions in children’s and adolescents’ physical activities, social interactions, and mental health [5].

There are likewise concerns regarding COVID-19’s adverse effects on children’s mental health due to the effects of changes in life and exercise habits caused by the pandemic. A study explained that children and adolescents showed symptoms of anxiety, depression, and stress due to school closures. Furthermore, only about 24% were satisfied with their lives [6]. Another study indicated that adolescents were more concerned about government restrictions for containing the spread of the virus rather than the virus itself. These concerns are associated with people’s increased anxiety and depressive symptoms and decreased life satisfaction [7]. Russell’s study showed that caregivers’ COVID-19 burden lead to depression, generalized anxiety, child stress, and parent-child relationship conflicts [8]. Even at home, conventional behaviors are suppressed by restrictions such as government lockdowns and social life limitations. Behavioral suppression brought about by COVID-19 has continued for more than a year. Therefore, it is necessary to consider its long-term effects [8]. According to a survey of elementary school students and their parents, approximately one-third of the parents reported being extremely anxious about COVID-19. During this period, most children increased television watching, using the computer and gaming. More than three-quarters of children used other screen-based devices. Unsurprisingly, approximately half of the children in the survey played less at the park and in public spaces given the mandated closure of playgrounds. On the other hand, children’s physical activities at home either increased or remained unchanged. Anxious parents’ children had fewer visits to the park and were more likely to spend 2 hours or more per day using the computer or gaming compared with less anxious parents’ children [9]. According to Rapee’s model, children’s anxiety is influenced not only by their own cognitive and behavioral factors, but also by their relationships with and support from friends, family, and society [10]. In Rapee’s conceptual model of social-emotional disorders, it is important to examine disorders’ impact on adolescents’ mental health. This is vital given the strong associations between adolescents’ interpersonal stress and emotional difficulties. Due to COVID-19’s influence, children’s relationships with other people and support resources mentioned in the model above are declining. Moreover, their self-formation is becoming more difficult. Therefore, it is important to have a complex understanding of environmental factors in modern situations. During the COVID-19 pandemic, it is difficult to have similar exchanges as before because direct interactions with friends and other people have become very limited. Before the pandemic, people often establish a self-concept and raise their self-esteem by interacting with friends or parents [11–13]. However, with the current situation, their range of activities is limited to some extent. In modern society, COVID-19’s effects limit children’s opportunities for education and behavioral learning at home. Furthermore, its impact on children’s mental health cannot be denied. Additionally, it may be difficult to acquire adaptive behaviors and skills because children’s behavioral restraints limit their social development and opportunities for interpersonal interaction.

Remote methods may be resorted to if direct psychological intervention is difficult to perform as a response to the resulting anxiety symptoms. Thus, cognitive behavioral therapy (CBT) over the Internet may be useful. As mentioned above, COVID-19 pandemic has a psychological effect on children, but considering the reduction of infection risk such as refraining from going out, it is necessary to utilize a remote method. Based on this, the usefulness of an Internet-based cognitive behavioral therapy (ICBT) is discussed in the following sections.

### ICBT’s applicability for anxiety-related problems in children

The usefulness of online CBT using the Internet can be seen when looking into the options for dealing with anxiety symptoms among children due to COVID-19. Particularly, face-to-face CBT has been shown to be effective. For example, a study compared the effectiveness of individual and group CBT for referred children with anxiety disorders within community mental health clinics. Among the children with anxiety disorders, both individual and group CBT can be effectively delivered in community clinics. The response rates were similar to those reported in the efficacy trials [14].

The application of self-help CBT is important in response to the growing anxiety on the infection risk brought about by face-to-face CBT implementation. Self-help CBT can provide a useful approach for psychological problems’ treatment. Self-help treatment such as computerized CBT (CCBT) can also solve problems in providing care for patients, such as the limited availability of clinicians trained in evidence-based interventions **[**15]. Additionally, CCBT is effective for patients who are reluctant to participate in treatment in clinical settings due to a certain stigma [16]. Self-help CBT programs have been used to improve depression, anxiety [17–19], and insomnia [20]**.** ICBT is a form of self-help CBT. It can be broadly divided into two types: self-help and therapist-assisted therapies. Self-help therapies can be implemented by the user at one’s discretion. However, its effect is generally moderate [21, 22]. Some therapist-assisted therapies are more effective, but the patients have more freedom in self-help therapies [23]. ICBT has traditionally been shown to be effective in psychotherapy, contributing to mental health care. Since ICBT is a tool for remote treatment, patients can obtain psychological intervention effects without going to a medical institution. The ICBT is not implemented in a face-to-face format. Thus, it is possible to access psychological intervention at a free environment, such as one’s home, in consideration of COVID-19’s impacts. Therefore, it is possible to obtain treatment remotely when it is difficult to go to a medical institution due to COVID-19.

A meta-analysis, twenty-five studies which targeted 11 different disorders were included in the review, reported that internet delivered CBT suggested large within-group effect sizes and moderate between-group effect sizes when compared with a waitlist in face-to-face therapy sessions. Sixty-four percent of the selected studies had the modules to conduct both parents and children. The other studies were conducted to only children. Nineteen of the studies were randomized controlled trials, two were quasi-randomized controlled trials and four were uncontrolled open trials. The results suggest that CBT for psychiatric and somatic conditions among children and adolescents can be successfully adapted into an Internet-based format. They indicated that further research is needed to evaluate for whom, when, and how such a delivery method for CBT is effective. Overall, present evidence supports the computerized delivery of CBT. They suggest that it is a promising alternative to clinical-based CBT, resolving the barriers to treatment access [24].

Some studies have also been conducted on ICBT’s effects. Jolsdedt’s study examined whether therapist-guided ICBT is feasible and potentially effective when implemented in an outpatient clinic in rural Sweden. Nineteen children aged 8–12 with anxiety disorders underwent a 12-week ICBT program called BiP Anxiety. Therapeutic gains were maintained for up to three months from the post-treatment assessment. At follow-up, 68% were no longer in need of treatment and could be discharged from the clinic. Overall, the participants and clinicians were satisfied with the treatment content and format. It was suggested that ICBT’s implementation could dramatically increase access to evidence-based treatment for children with anxiety disorders who live far away from specialist clinics **[**25]. Another study performed 10-week ICBT. Participants were 90 children aged 8 to 12 years who had diagnostic criteria for anxiety disorders. The results showed that the improvement was significant and ICBT had a high effect size. The other randomized controlled trial was conducted to evaluate ICBT’s effectiveness among children with anxiety disorders. Participants were divided into two groups and conducted 10 weeks of ICBT with therapist support or a waitlist control condition. The primary outcome measure is Clinician Severity Rating (CSR), that is derived from the Anxiety Disorder Interview Schedule Child and Parent version [26]. There was a large between-group effect size post-treatment of the measure. Parent-reported child anxiety was significantly lower in the treatment group than in the waitlist group post-treatment. And it was also reported that there was a small between-group effect size. Spence’s research examined social support’s role in predicting treatment adherence and outcomes among youth enrolled in an open-access ICBT intervention that targets anxiety. They studied over 3000 children aged 7 to 17 years who were reported to have elevated levels of anxiety symptoms. They showed that participants with greater social support from all sources showed greater program adherence. Age moderated the effect of family support upon adherence. Specifically, greater family support was associated with more sessions completed only for older youth. Greater families and overall support were associated with greater anxiety relief, regardless of participant age. Younger participants were more likely to complete more sessions and show greater improvement of anxiety. The findings underscore the need to consider ways to adhere to treatment and enhance outcomes among children engaged in self-help ICBT for anxiety disorders. These results indicated that the necessity to consider how to enhance the adherence of treatment program and outcomes among children engaging in self-help ICBT for anxiety problems. These are important especially when social support is low [27]. Other study investigated that fifty-two children aged 3–6 years were randomly allocated into internet-based CBT and waitlist control groups. Parents completed diagnostic interviews and online questionnaires at pre-treatment, post-treatment and 6- month follow-up. Its results at post-treatment showed a significantly greater reduction in clinical severity, anxiety symptoms and internalising behaviour, as well as a greater increase in overall functioning for children in the internet treatment group compared to the waiting list condition. At post-treatment for the completer sample, 39.1% of the CBT group of children compared to 25.9% of the waiting list group were free of their primary diagnosis. By 6-month follow-up for the completer sample, 70.6% of children were free of their primary diagnosis. The results suggest that an internet program for preschool anxiety is feasible, efficacious and well received by parents [28]. Another study examined the effect of internet-delivered parenting program. They investigated the effect of the predictors of child anxiety outcomes of about 400 families with young children (3–6 years) who participated in a randomized controlled trial which is eight-module early intervention program for child anxiety based on CBT. The results showed that only printer access moderated intervention effectiveness. Printer access, which means that participants have printing equipment in their houses, predicted lower child anxiety in the Online CBT group. However, it had no effect on the outcomes in the waitlist group. As a result of further examination, it was indicated that higher levels of child anxiety at baseline, child’s suppressed temperament, and worse parental mental health resulted in higher child’s anxiety in both groups at 6-month follow-up period. Parents who reported practicing the program skills more frequently showed greater reductions in child anxiety. These findings provide empirical support for skills practice’s important role in ICBT interventions. Furthermore, they suggest that practicing program skills may be more important than completing online modules. A previous study described in detail the use of computers as a medium for delivering specific aspects of interventions **[**29]. A trial examined the relative efficacy of online versus clinic CBT delivery in the treatment of adolescents’ anxiety disorders. About 110 participants and their parents were randomly assigned to online group, clinic CBT group, or waitlist control conditions. The treatment groups received equal CBT content. The assessment at post-baseline showed significantly greater reductions in anxiety diagnoses and anxiety symptoms for both CBT treatment conditions compared with the waiting list group. These improvements were maintained or further enhanced for both conditions at the 6- and 12-month follow-ups, with minimal differences between them. About 80 % of adolescents in the online group of completer participant no longer met the criteria for the principal anxiety diagnosis at the 12-month follow-up [30].

**Fig. 1:**
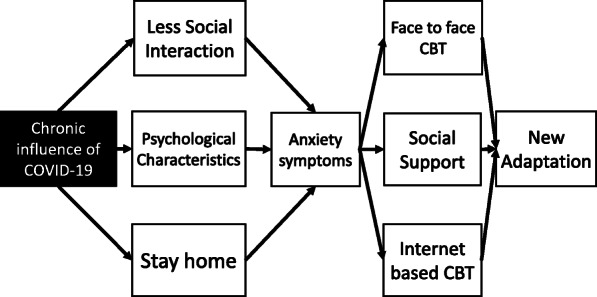
Conceptual model of anxiety symptoms during the COVID-19 pandemic

An overview of these studies shows that ICBT has a certain effect on child anxiety. Studies have shown equal or slightly inferior effects on face-to-face CBT. However, it is a more useful tool in modern situations given the difficulty in practicing face-to-face psychotherapy due to COVID-19’s effects. In this model, we constructed and proposed a conceptual model based on previous studies and COVID-19’s current status. Anxiety symptoms under the context of COVID-19 are thought to be affected by prolonged stay homes, decreased direct social interaction, and individual psychological characteristics. For these improvements, the establishment of face-to-face CBT and ICBT as much as possible, as well as appropriate social support, will facilitate adaptation in the current situation. Since the impact of COVID-19 is prolonged due to the presence of delta and omicron strains, it is considered necessary to use internet-CBT, expand online social support, and maintain conventional social activities to the extent possible after taking countermeasures, Fig. 1.

## Discussion and conclusions

This study reviews the effects of COVID-19 on children’s mental health. It likewise explores the effects of ICBT on children’s anxiety symptoms. COVID-19’s effects are still ongoing, and it has been clarified that this pandemic has long-term psychological and physical effects on people. It has been shown in previous studies that that COVID-19 increases the burden not only for children but also for parents and caregivers. It can be said that a system that can provide sufficient care is necessary considering COVID-19’s long-term effects because of depressive symptoms, anxiety, and stress are worsening as psychological effects. Therefore, while the mental health measures for anxiety and related problems continues, it is necessary to continue the life called New Normal and take measures focusing on children’s future lives. For example, ICBT has been effective in dealing with anxiety and other problems. Additionally, it has been pointed out that there are some issues regarding depression and physical health. Thus, this tool is expected to be applied to many problems as well, not only for COVID-19 complications. Moreover, children’s developmental concerns are widespread. Sufficient support is provided not only for mental health problems such as anxiety due to restrained life in prolonged situations. Although ICBT focuses on improving mental health problems, it can be said that future development is expected in terms of child development, interaction with friends, and acquisition of social skills. At present, there is a possibility that ICBT in a group can also be developed in the future when online tools become better. Globally, remote learning is affected by nations’ economic situations. It cannot be denied that low-income countries do not have a sufficient environment for online learning. For example, a meta-analysis indicated that the children in the reviewed studies were from mid- to high-income families. This is a confounding variable in studies where internet and ICBT services are being used **[**23]**.** Future studies may recruit from a wider socio-economic background to disentangle these factors given that computer ownership and access to a home broadband connection are rapidly spreading globally. The current review suggests that children with primary anxiety disorders may benefit from CBT programs that include computerized delivery. Regarding ICBT, it is possible to establish a system in any economically developed country. Thus, international support may be required to eliminate this disparity among countries. ICBT is considered useful in supporting mental health. However, there are some limitations when it comes to using only the Internet for treatment. Obtaining information and making assessments of details such as behavioral observations and body language may be difficult, unlike in face-to-face interviews. Thus, it is necessary to stabilize the communication status on the Web and add realistic exposures and face-to-face assistive interviews to resolve these issues. Further effectiveness can be achieved by expanding the response to anxiety-related problems and methods in supporting children. Additionally, in the case of ICBT through the Internet, it is also necessary to confirm in advance the emergency measures to be taken in case of sudden physical or mental disorders. In addition, one review discusses the application of SAD exposure for youth in teletherapy [31]. In this review, they argued that managing their embarrassment, leaning into the challenges, and creatively identifying unique and relevant exposures that elicit the same core fears as the more “tried and-true” social exposures, are essential skills to the successful treatment of social anxiety in youth. A variety of beneficial attempts to improve children’s mental health may be necessary under the influence of COVID-19.

## Data Availability

Not applicable.

## Abbreviations

CBT: cognitive behavioral therapy
CCBT: computerized cognitive behavioral therapy
COVID-19: coronavirus disease 2019
ICBT: Internet-based cognitive behavior therapy
RCT: randomized controlled trial
